# Validation of the severe respiratory insufficiency questionnaire for Chile

**DOI:** 10.1186/s12890-022-02050-7

**Published:** 2022-07-19

**Authors:** Marianela Andrade, Mónica Antolini, Krishnna Canales, Cesar Maquilon, Mauricio Fuentes, Marinella Mazzei

**Affiliations:** 1 AVNIA-AVIA Programs, Ministry of Health, Santiago, Chile; 2 Quality and Accreditation Clínica Dávila and AVNIA-AVIA Programs, Ministry of Health, Clínica Dávila, Santiago, Chile; 3 Department of Respiratory Diseases, Recoleta 464, Building H, 6th floor, Santiago, Chile; 4 School of Public Health, Universidad de Chile, Santiago, Chile

**Keywords:** Questionnaire validation, Quality of life, Home mechanical ventilation, Psychometry

## Abstract

**Background:**

Long-term home non-invasive ventilation (LTH-NIV) has an impact on the health-related quality of life of patients with chronic hypercapnic respiratory failure (CRF) of different causes. There are generic and specific questionnaires for respiratory diseases. In 2003 a specific questionnaire was developed for patients with CRF in LTH-NIV, called the Severe Respiratory Insufficiency (SRI) questionnaire, which has been shown to be reproducible and reliable and has been validated in several languages.

The aim of the study was to translate and culturally adapt the SRI questionnaire for adult Chilean patients under LTH-NIV, and to assess its psychometric properties.

**Methods:**

The Chilean version of the SRI was obtained using the translation-back translation method, which was then applied by cross-sectional study to a non-probabilistic convenience sample of stable patients from five regions of Chile. The validated Chilean version of the SRI questionnaire and SF-36 (gold standard) questionnaire were applied, demographic and ventilatory data were collected. Reliability was analysed using Cronbach’s alpha and intraclass correlation (test–retest). Construct validity was tested using exploratory factor analysis (principal component extraction and equimax orthogonal rotation) and hypothesis testing (Mann–Whitney test). Convergent criterion validity was tested using Spearman’s rho.

**Results:**

The sample comprised 248 patients, 132 women (53.2%), median age (IQR) was 62 years (51–75), 146 patients (58.9%) were 60 years or older, 40% had a low education level. The mean ± SD completion time of the questionnaire was 18.8 ± 9.1 min, and 100% of the items were answered. The questionnaire was self-applied by 46.8% of the sample. The validated Chilean version of the SRI questionnaire showed very good overall reliability (0.95) and by scales (> 0.7). It showed a good correlation with the SF-36, with equivalent scales, a rotated matrix with 8 factors and hypotheses that explain the underlying constructs.

**Conclusions:**

The validated Chilean version of the SRI questionnaire has good psychometric properties. It is feasible, valid, and reliable for application to evaluate patients with CRF in LTH-NIV. It was found to be sensitive to assess the characteristics of the local population.

**Supplementary Information:**

The online version contains supplementary material available at 10.1186/s12890-022-02050-7.

## Background

In Chile, National Health Surveys conducted in 2003, 2010 and 2017 showed that over the last two decades, the prevalence in adults of obesity and smoking has increased significantly, thus the prevalence of obesity increased from 21.9% in 2003 to 33.4% in 2017, the prevalence of smoking in 2003 was 43.5% and managed to drop to 33.3% in 2017. [1–3]. This contributed to an increased prevalence of Chronic obstructive pulmonary disease (COPD) and obesity hypoventilation syndrome (OHS) [4, 5]. When these patients develop CRF are added to the list of diseases that benefit from LTH-NIV, which include neuromuscular diseases and kyphoscoliosis.

The LTH-NIV has an impact on the health-related quality of life (HRQL) of these patients, and although there are generic questionnaires that can be applied to them, such as the Sickness Impact Profile [6] and 36-item Short-form Health Survey (SF-36)[7], or specific questionnaires for the most prevalent respiratory diseases, such as chronic obstructive pulmonary disease (COPD)–St. George’s Respiratory Questionnaire (SGRQ) [8], Chronic Respiratory Disease Questionnaire [9], and London Chest Activity Daily Living [10, 11]–these questionnaires are not sensitive for the specific group of patients on LTH-NIV.

The Severe Respiratory Insufficiency (SRI) questionnaire, developed in Germany in 2003 [12], responds to the need of monitoring changes in HRQL in adults with LTH-NIV. It was developed with a solid methodological design and has good psychometric properties. It has been translated and validated in different languages in European and Asian countries as part of the SRI Project [13]. The only translation of the original SRI into Spanish was made in Spain (Lopez-Campos MD) [14], and as usual it is the one used in Latin America.

Chile has had a public home mechanical ventilation program for children since 2006 and one for adults since 2008 (Additional file 1. Home mechanical ventilation technical standard -versions- 2008- 2012-and-2013, and informed consent. Chile.doc). So, it was pertinent to validate the SRI instrument for Chile to avoid the lack of equivalence when using an external version (SRI Spain), improve the analysis of results and collaborate in the development of health objectives. Patients belonging to the Chilean state home mechanical ventilation programs probably have a different educational level, suffer from numerous comorbidities and their economic situation may be different from that of Spanish or English patients and others [15], so the validation carried out in these countries does not necessarily represent our universe of patients. This situation probably is similar in other Latin-American countries. For this it was necessary to carry out the translation and cultural adaptation of the SRI questionnaire to Chilean patients, following the translation/back-translation method [16–19], obtaining an equivalent version to the original.

## Methods

### The SRI questionnaire

The SRI is a questionnaire self-administered by LTH-NIV users (non-tracheostomized) that consists of 49 items (statements), grouped into seven dimensions (scales) that the subject rates according to their perception considering their state of health during the last week. It has a five-point Likert scale from “completely untrue” to “always true”. The seven dimensions are respiratory complaints (RC); physical functioning (PF); attendant symptoms and sleep (AS); social relationships (SR); anxiety (AX); psychosocial well-being (WB) and social functioning (SF).

Once the instrument is filled out, of its 49 items, 35 are recoded by inverting their value to leave all the items in the same direction. After recoding these items, the score of each dimension is obtained according to a mathematical transformation that uses in the numerator the mean value of the scores obtained for the items of the dimension minus 1, divided by the maximum distance of the Likert scale, that is, divided by four. The dimensions are expressed in percentages (0 to 100%). Higher scores are attributed to better HRQL.

### Translation and back-translation process

The Chilean SRI was the result of transcultural adaptation, this included translation and back-translation of the original German SRI and validation suggested by Hispanic American guidelines [20–22]. An expert committee was formed with cardiorespiratory health professionals selected for their expertise and background (two were bilingual Spanish-German doctors). Four translators recommended by the German Embassy in Chile (19) participated (two Chilean and two German, both native language speakers), who worked in parallel and blindly without communication with each other. An editor in charge and fieldwork coordinator was also selected.

The back-translation version was reviewed by the original author (Winsdich W. MD) to qualify the semantic differences with the original SRI and those found were adjusted until “full equivalence” items were achieved.

### Semantic validation

A pilot test was carried out to study the understanding of the Chilean SRI achieved. A convenience sample of 15 patients with CRF in LTH-NIV, in a stable phase of their disease, with different educational levels (who can read and write) were selected so that they could make corrections, comments and suggestions on any section that hindered their understanding of the test. The items that were difficult to understand in 15% of the interviewees, would be reviewed to consider the suggested modifications [20–22].

The pilot was executed in a standardised way by 2 professionals with experience in applying the test, the information obtained from the subjects was tabulated and consolidated by the first author of the study [(Additional file 2. Semantic validation of the SRI quality of life questionnaire 2021 Article Protocol SRI.1.doc) (Additional file 3. Semántica Validación en Español del Cuestionario de Insuficiencia Respiratoria Severa en CHILE. Artículo Protocolo IRS 1.0.docx].

**Fig. 1:**
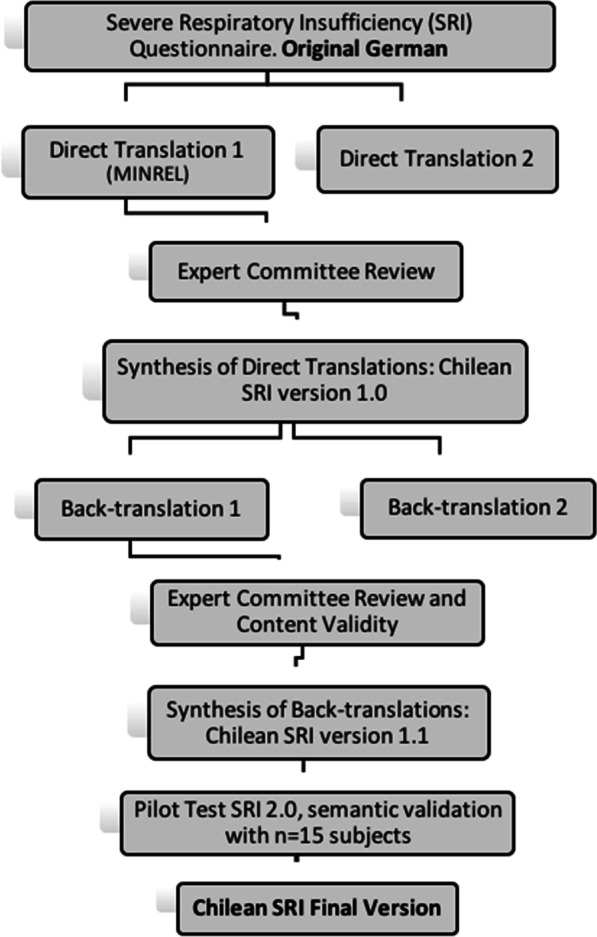
Chilean SRI translation and back-translation process

A summary of the translation and back-translation process is shown in Fig. 1.

### Psychometric evaluation: study design and sample

This was a cross-sectional study with a non-probabilistic convenience sample of patients with CRF on LTH-NIV belonging to the Program of HMV of the Ministry of Health of Chile, from five regions of the country. Patients with cognitive impairment that prevented understanding the instructions, patients experiencing cardiorespiratory exacerbation and users of continuous positive airway pressure (CPAP) or invasive mechanical ventilation (SRI is not validated for them), were excluded. A sample size of at least 245 patients was estimated for an adequate factor analysis (5 patients for each item of the instrument) [20–23]. Participants were interviewed at home in person by a nurse, trained for the activity, who were impartial and tasked with obtaining good quality records. Two questionnaires were administered on the same day: the Chilean SRI version and the SF-36, their completion time was recorded. Sociodemographic and clinical data were also collected.

### Statistical analysis

A sociodemographic analysis of the sample and description of the items and dimensions were performed by calculating measures of central tendency and dispersion. Reliability was evaluated using Cronbach’s alpha [20, 21], measuring the correlation of each item with the total score of the questionnaire and of each item with the total score of its scale, expecting to obtain coefficients > 0.70 [17, 18]. In addition, the test–retest reliability was evaluated by applying the same Chilean SRI version at two different times (baseline and one week later) to a subgroup of the sample comprising 20 patients, calculating the intraclass correlation coefficient (ICC), expecting to obtain ICC > 0.8 with a confidence interval (CI) of 95%.

### Construct validity

To evaluate whether the different groups of items of the Chilean SRI adequately represented the dimensions of the underlying theoretical construct, two analyses were performed: (i) Exploratory factor analysis (EFA), and (ii) Simple hypothesis testing.

(I) In the EFA, the following measures of sample adequacy were assessed: Kaiser–Meyer–Olkin (KMO), expecting an adequate value ≥ 0.70; Bartlett’s test of sphericity; and principal components extraction, with the Kaiser rule stating that all factors with eigenvalues > 1 should be retained [24, 25]. To facilitate interpretation orthogonal rotation was applied (varimax and in the face of difficulty in interpretation equimax to simplify the factors and items) [24–27].
(II) The hypotheses proposed were based on the fact that HRQL is differently affected according to the underlying disease [13, 14]. The five are as follows: (i) Patients with COPD have a lower score on the Respiratory Complaints (RC) dimension than patients without COPD; (ii) Patients with respiratory failure of obstructive origin have more respiratory complaints (RC) than those with respiratory failure of restrictive origin; (iii) Patients with COPD are more affected by anxiety (AX) than patients without COPD; (iv) Patients with neuromuscular disease have lower scores on the Physical Functioning (PF) scale than patients with non-neuromuscular disease; and (v) Patients who are connected more hours a day to NIV have worse quality of life. The Mann–Whitney test (i–iv) and Spearman’s correlation (v) were applied.

### Criterion validity

The Chilean version of the SRI was compared with the generic questionnaire SF-36 (gold standard in HRQL and validated in Chile) as an external criterion to evaluate convergent validity [28, 29]. The SF-36 has 36 items distributed across eight scales: Physical Functioning, Physical Role Functioning, Bodily Pain, General Health Perceptions, Vitality, Social Role Functioning, Emotional Role Functioning and Mental Health. It was used in the original and European validations of the SRI, correlating its results according to the equivalent subscales, using Spearman’s rho correlation coefficient [16, 22]. High correlation was expected between the homonymous and conceptually related dimensions, and low correlation was expected for the unrelated dimensions (e.g., Bodily Pain versus Attendant Symptoms and Sleep).

The study protocol was approved by the ethics committee of the North Metropolitan Health Service (Nº 004/2016), an informed consent was signed by all participants, and the data obtained were kept confidential by the principal investigator. The statistical software SPSS version 21 was used for all the analysis.

## Results

### Cultural adaptation, translation, and semantic validation

The translation was rated with a low level of difficulty and a high level of naturalness (score 2 and 9, respectively). The Likert scale translated as “totally false” to “totally true” was rejected by the panel of experts, because this format was already known in Chile (through the application of SRI Spain) and in practice it presented difficulties in the understanding of patients. The expert committee established a new Likert scale whose options range from “totalmente en desacuerdo [strongly disagree]” to “totalmente de acuerdo [strongly agree]”.

In the back translation version, the equivalence of items between the Chilean SRI and the original German, (qualified by the original author Dr. Winsdich) determined as “A: totally equivalent” 19 items, “B: similar” 27 items and “C: doubtful or not equivalent” 3 items. Finally, the adjusted version of the German back translation was resubmitted, and the author approved all 49 items.

About semantic validation, the pilot test participants completed the questionnaire in a (mean ± SD) time of 14.5 ± 6 min, and all reported good understanding of the introductory text. In relation to the 49 items, only 5 items needed to be modified (items 9, 14, 17, 19 and 30). The use of the expanded Likert scale as a visual aid was accepted and considered useful. The full version of the questionnaire is attached at the link (Additional file 2. Semantic validation of the SRI quality of life questionnaire 2021 Article Protocol SRI 1.0.doc).

### Psychometric validation

The Chilean version of the SRI and SF-36 questionnaires were administered to 268 patients, all with CRF in stable phase. Twenty questionnaires were excluded for not completing the Chilean SRI, nineteen of them for not answering item 31 “my marriage/relationship is suffering because of my illness”, corresponding to patients without a partner. Finally, the valid sample with 100% of the items answered comprised 248 patients, of whom 132 were women (53%), aged (mean ± SD) 62 ± 17 years in a range from 20 to 88 years old, thus predominantly older adults. Forty percent of the sample had a low educational level (incomplete primary education 36%; no schooling 4%). Fifty one percent of the sample was in a relationship (48% married, 3% cohabitating). The sample was grouped into eight diagnostic groups: (1) COPD; (2) COPD and obstructive sleep apnea (COPD-OSA); (3) Tuberculosis sequelae (TB); (4) non-cystic fibrosis bronchiectasis (non-CF BE); (5) neuromuscular diseases (NMD); (6) Kyphoscoliosis (KYPH); (7) Obesity hypoventilation syndrome (OHS); and (8) Various disorders. All patients enrolled had severe CRF, the demographic and functional characteristics were described in Table 1. A total of 170 patients (69%) used continuous oxygen therapy, 35% of them 24 h a day. Regarding smoking, 61% reported stopping smoking (for at least 6 months), and 2% reported active smoking; 51% of the COPD patients were GOLD 4 and 38% GOLD 3.

**Table 1:** Demographic and functional characteristics of the sample studied (n = 248 patients)

	COPD	COPD-OSA	TB	Non- CF BE	NMD	KYPH	OHS	Various	All patients
n (%)	66 (26.6)	34 (13.7)	8 (3.2)	18 (7.3)	28 (11.2)	27 (10.9)	60 (24.2)	7 (2.8)	248
Age in years (median ± SD)	71 ± 8.2	67 ± 10.7	67 ± 8.7	46 ± 15.1	24 ± 15.2	54 ± 20.8	61 ± 11.6	59 ± 21.6	62 ± 17.5
Sex (male/ female)	33/33	18/16	3/5	11/7	17/11	15/12	18/42	1/6	116/132
Months on NIV (mean ± SD)	29.7 ± 25.6	41.4 ± 25	44.5 ± 27.1	31.0 ± 22.1	55.1 ± 42.6	39.0 ± 27.1	37.2 ± 26	30.0 ± 16	37.6 ± 28.5
Use of NIV h/day (mean ± SD)	7.4 ± 2.3	7.5 ± 1.7	8.3 ± 1.4	8.4 ± 2	8.1 ± 3.2	8.4 ± 3.9	7.0 ± 1.4	7.8 ± 1.1	7.6 ± 2.4
FVC % pred (mean ± SD)	58.8 ± 21.5	63.4 ± 16.3	58.2 ± 9.3	47.0 ± 21.5	36.9 ± 18.9	38.7 ± 14.1	70.5 ± 18.0	46.8 ± 15.9	57.1 ± 21.6
FEV_1_% pred (mean ± SD)	33.5 ± 17.2	49.5 ± 18.5	40.3 ± 6.1	34.4 ± 21.4	38.0 ± 17.5	35.0 ± 10.9	68.1 ± 20.1	35.8 ± 11.4	45.5 ± 22.2
FEV_1_/ FVC % (mean ± SD)	45.5 ± 15	61.6 ± 15.3	55.6 ± 9.2	56.1 ± 12.6	89.3 ± 8.6	78.2 ± 13.8	77.9 ± 10.3	65.7 ± 21.2	65.3 ± 20
pH (mean ± SD)	7.36 ± 0.04	7.36 ± 0.04	7.35 ± 0.03	7.38 ± 0.03	7.38 ± 0.03	7.37 ± 0.05	7.37 ± 0.01	7.36 ± 0.01	7.36 ± 0.04
PaCO2 (mean ± SD)	59.8 ± 9.4	61.7 ± 11.6	60.2 ± 6.3	59.5 ± 5.4	57 ± 23.3	57.3 ± 9.7	58.9 ± 10.5	59.3 ± 13.9	59.3 ± 11

*COPD* chronic obstructive pulmonary disease, *OSA* obstructive sleep apnea, *TB* pulmonary tuberculosis sequelae, *Non-CF BE* Non-Cystic Fibrosis Bronchiectasis, *NMD* neuromuscular disease, *KYPH* Kyphoscoliosis, *OHS* hypoventilation obesity syndrome, *FVC* forced vital capacity, *FEV*_*1*_ forced expiratory volume in 1 s; *PaCO*_*2*_ partial pressure of carbon dioxide, Blood arterial gases baseline daytime (room air or oxygen supply in dependent patients needing supplementary oxygen) values were collected before entering the HMV program

The completion time of the Chilean version of the SRI (mean ± SD) was 18.8 ± 9.1 min. It was self-administered in 46.8% (n = 116) of the patients, and the remaining 53.2% required interviewer assistance, reporting difficulty in reading and writing due to low educational level, severe dyspnea, essential hand tremor and visual difficulty. In these cases, the interviewer used an enlarged printed Likert scale as an aid for the patients to indicate their response.

### Analysis of the instrument items

The distribution of the raw responses showed that the Chilean SRI items were heterogeneous and had discriminatory capacity. The item-scale correlations between the 49 items and their theoretical dimensions were significant and direct (positive), with rho values > 0.40, being higher than the correlations with other scales (Table 2).

**Table 2:** Summary of item-scale correlations of the Chilean SRI using Spearman’s correlation coefficient

Chilean SRI dimensions	Number of items	Correlation range of the items with their theoretical scale	Correlation range of the items with other scales
Respiratory complaints (RC)	8	0.59–0.84*	0.20–0.57
Physical functioning (PF)	6	0.50–0.79*	0.02–0.68
Attendant symptoms and sleep (AS)	7	0.45–0.69*	0.04–0.58
Social relationships (SR)	6	0.52–0.74*	0.09–0.63
Anxiety (AX)	5	0.61–0.75*	0.11–0.55
Psychosocial well-being (WB)	9	0.49–0.74*	0.16–0.56
Social functioning (SF)	8	0.46–0.74*	0.19–0.64

*p-value < 0.01. Range expressed as minimum—maximum

### Reliability

The Chilean SRI presented very good overall reliability (0.95), higher than the original SRI (0.89). Its seven theoretical dimensions showed good internal consistency, between 0.71 and 0.84. The lowest alpha was obtained for Attendant Symptoms and Sleep, and the highest one for Respiratory Complaints. The alphas by dimension were similar between Chilean SRI and the original SRI, indicating that both measure their constructs in a similar way. The ICC was very good (> 0.8) in the seven dimensions, indicating excellent agreement and temporal stability of the instrument at both times (Table 3). At this stage, it was not indicated to change or exclude any item from the theoretical dimensions.

**Table 3:** Internal consistency according to subscale by the intraclass correlation coefficient (ICC) and Cronbach’s alpha

SRI dimensions	Number of items	ICC (95% CI) SRI Chile	Cronbach’s α SRI Chile	Cronbach’s α SRI Germany	Cronbach’s α SRI Spain
Respiratory complaints (RC)	8	0.84 (0.63–0.94)	0.84	0.83	0.81
Physical functioning (PF)	6	0.94 (0.85–0.98)	0.73	0.80	0.82
Attendant symptoms and sleep (AS)	7	0.91 (0.78–0.96)	0.71	0.76	0.73
Social relationships (SR)	6	0.92 (0.80–0.97)	0.72	0.73	0.63
Anxiety (AX)	5	0.95 (0.87–0.98)	0.78	0.79	0.73
Psychosocial well-being (WB)	9	0.93 (0.83–0.97)	0.82	0.89	0.85
Social functioning (SF)	8	0.91 (0.77–0.96)	0.80	0.84	0.82
SRI global (summary scale)	49	0.97 (0.92–0.99)	0.95	0.89	0.93

### Construct validity assessment by exploratory factor analysis

The measures of adequacy of the sample were favourable to perform an exploratory factor analysis (K.M.O. index > 0.90 considered very good), indicating a significant correlation between items. There were no items with low communality (< 0.30), the communality range was between 0,45–0,76. Only items 7, 9, 12 and 23 obtained communality < 0.50 and were considered under observation for the final analysis.

Exploratory factor analysis, according to the Kaiser criterion, gave a solution of 12 factors with eigenvalues > 1.0, explaining 64.5% of the total variance. However, this analysis suggested, according to the percentage of variance criterion, a model with only 2 factors but this is not convenient because of their low significance in the weightings (36.31%), and so the use of the eigenvalue > 1.0 criterion was prioritised.

**Fig. 2:**
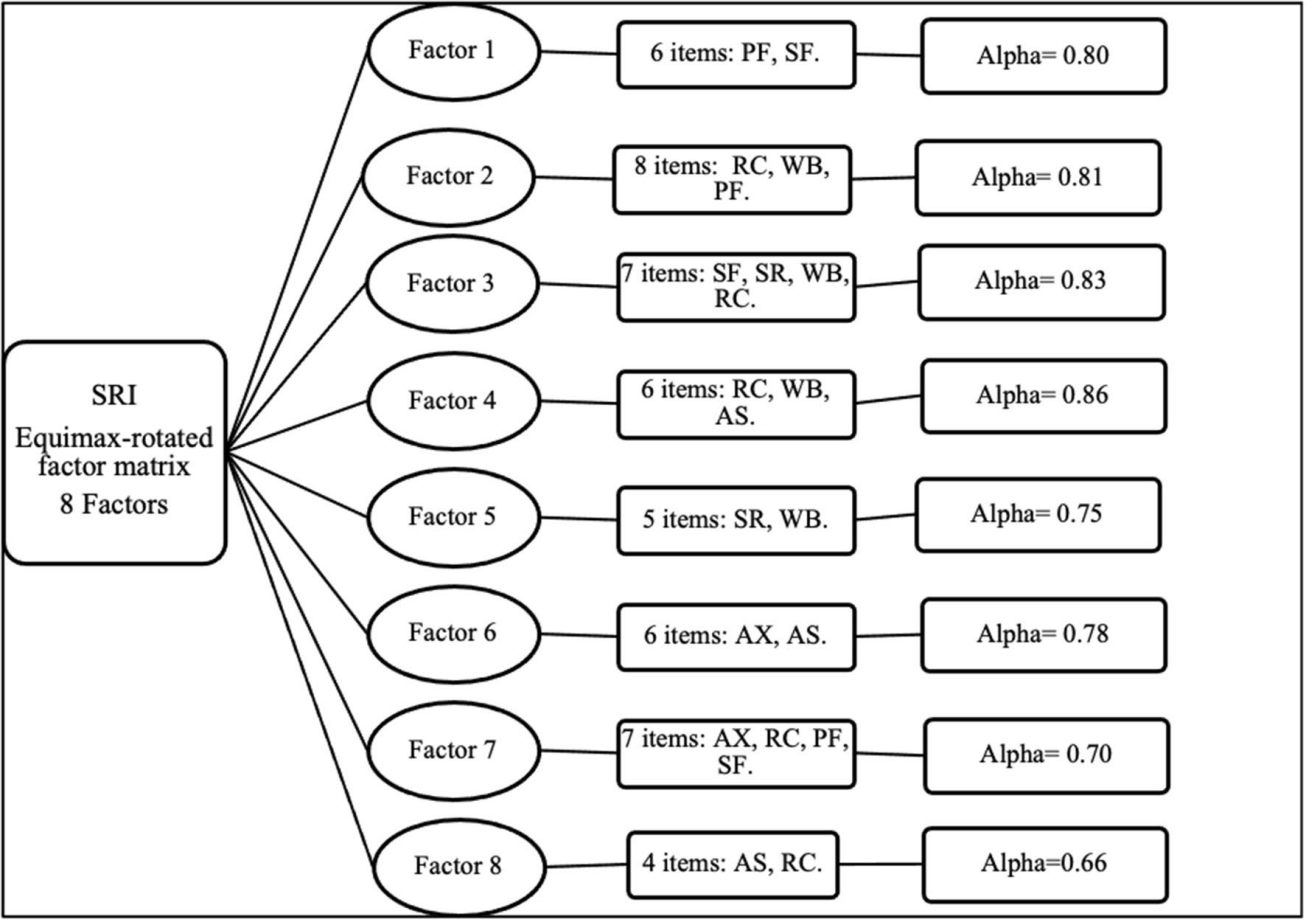
Schematization of the factors extracted from the Chilean version of SRI with equimax orthogonal rotation, The SR dimensions are presented: Respiratory Symptoms; FF: Physical function; SS: Accompanying symptoms and sleep; SR: Social relations; AX: Anxiety, BP: Psychosocial Well-being; FS: Social function

To clarify the structure of the factors in the matrix and optimise the differentiation of the items, orthogonal rotations were applied. First, varimax was used, but it did not achieve a completely satisfactory model when extracting 6 to 10 factors. It then decided to use equimax to simplify the factors, finding a solution of 8 factors explaining 55.6% of the total variance, which satisfactorily simplified the model. The factors with the items showed a good correlation. Conceptually, they demonstrated that all the constructs of the original instrument are present and that they are relevant for patients with CRF (Fig. 2). Although the factors found do not conform identically to the dimensions of the theoretical instrument, their organisation in the Chilean sample is consistent.

Modifying the structure of the Chilean SRI subscales based on the EFA was rejected, since when performing the reliability analysis of the new factors, the Cronbach’s alpha values decreased with respect to the original 7-scale structure.

### Construct validity assessment by hypothesis testing

Four out of the five hypotheses were consistent with the results. The worst scores (expressed as medians) on the RC dimension were observed for COPD = 53 versus neuromuscular disease = 75 (*p* < 0.001), versus Kyphoscoliosis = 69 (*p* < 0.003) and versus OHS = 69 (*p* < 0.002). In addition, on the RC dimension, the group of patients with obstructive disease (n = 129) scored worse than the group with restrictive disease, 53 versus 69 (*p* < 0.001). Additionally, in the group of patients with obstructive disease, there was no significant difference in scores on the RC among the diagnostic subgroups, as expected: COPD = 53 versus TB = 44 (*p* = 0.65) and versus BE = 58 (*p* = 0.50).

On the AX dimension, COPD = 40 was more affected than neuromuscular disease = 68 (*p* < 0.001) and Kyphoscoliosis = 55 (*p* < 0.03). Conversely, there was no difference in the AX subscale score in the group of patients with obstructive disease: COPD = 40 versus TB = 35 (*p* = 0.99) versus BE = 45 (*p* = 0.80).

Regarding the PF dimension, the score of patients with Neuromuscular versus Non neuromuscular disease showed no significant difference (*p* = 0.60), contrary to expectations. This may be explained by the severe deterioration in lung function of the sample (higher of PaCO_2_ levels) at the time of start of LTH-NIV compared to European patients.

The correlation between hours of ventilation/day and quality of life was negative and significant only for the PF dimension (rho = − 152 and *p* = 0.05), and according to the diagnostic group, it was only significant for COPD (rho = − 0.198 *p* = 0.024).

### Criterion or convergent validity

According to Table 4, the Chilean version of the SRI showed positive and significant (*p* < 0.01) correlations with the SF-36 v2 in its related dimensions, which were considered good. The strongest correlations were observed for PF, WB, and SF.

**Table 4:** Convergent validity between the dimensions of the Chilean SRI questionnaire and general quality of life measured by the SF-36 v2 (n = 248)

Chilean SRI questionnaire	SF-36v2 questionnaire							
	Physical functioning	Physical role functioning	Bodily pain	General health perception	Vitality	Social role functioning	Emotional role functioning	Mental health
Respiratory complaints	0.39	**0.50**	0.29	**0.57**	0.44	0.46	0.44	0.42
Physical functioning	**0.77**	**0.67**	0.30	**0.55**	**0.56**	**0.55**	**0.57**	**0.53**
Attendant symptoms and sleep	0.35	0.44	0.35	0.46	0.44	0.38	0.43	0.49
Social relationships	0.40	0.47	0.22	0.40	**0.55**	0.48	0.49	**0.57**
Anxiety	0.33	**0.50**	0.27	**0.56**	0.45	0.42	0.43	0.43
Psychosocial well-being	0.45	**0.55**	0.31	**0.64**	**0.70**	**0.57**	**0.59**	**0.77**
Social functioning	**0.53**	**0.61**	0.33	**0.61**	**0.62**	**0.59**	**0.59**	**0.58**
Total SRI summary scale	0.57	0.67	0.37	0.69	0.68	0.62	0.64	0.67

Spearman’s rho correlation coefficient

The correlation is significant at the 0.01 level (one-tailed) for all dimensions

Correlations > 0.50 are bolded, and the highest correlations (> 0.60) are bolded and underlined

### General results of the Chilean version of the SRI

The studied sample showed that patients with CRF on LTH-NIV perceived their HRQL as fair, with a summary scale score of 57%, with greater deterioration in HRQL observed in women (51%).

The Chilean SRI dimensions with the worst score were Anxiety with 45% and Physical Functioning with 50% and those with the best score were Social Relationships with 65% and Respiratory Complaints with 63% (Table 5). In the seven dimensions, women presented a worse perception of HRQL than men.

**Table 5:** Results of the Chilean SRI according to diagnostic group and instrument dimension

	Obstructive				Restrictive				
SRI dimensions	COPD	COPD-OSA	TB	Non-CF BE	NMD	KYPH	OHS	Miscellaneous	All Dg
Respiratory complaints	50	59	44	58	75	69	69	72	63
Physical functioning	38	58	38	56	46	63	54	42	50
Attendant symptoms and sleep	46	50	50	63	66	64	61	57	57
Social relationships	58	58	77	67	83	67	63	67	65
Anxiety	40	48	35	45	68	55	40	70	45
Psychosocial well-being	53	59	57	55	64	58	55	61	56
Social functioning	38	55	49	53	74	63	53	69	53
Total SRI summary scale	48	56	48	57	66	64	57	63	57

*COPD* chronic obstructive pulmonary disease, *OSA* obstructive sleep apnea, *TB* pulmonary tuberculosis sequelae, *Non-CF BE* Non-Cystic Fibrosis Bronchiectasis, *NMD* neuromuscular disease, *KYPH* Kyphoscoliosis, *OHS* hypoventilation obesity syndrome, *DG* diagnostic groups. Results expressed as the median. Total SRI Summary Scale values in bold

The analysis of psychometric properties did not eliminate items or modify the theoretical dimensions.

## Discussion

The present protocol has as strengths the application of the questionnaires by professionals in person at the patients’ homes. There were no missing items (100% response), unlike when questionnaires are sent by correspondence to patients [30].

The Chilean sample (n = 248) was larger than the original German sample (n = 226) [12] and other European samples (SRI Spanish, n = 115; British n = 152; Portuguese, n = 93)  [14, 31, 32], strengthening the factor analysis. A portion of the respondents in the present sample was socially vulnerable, as expressed by their low education level, predominance of older age and greater percentage of work disability, parameters that indicate further deterioration in health status compared to the Spanish group [15, 33].

The main difference between the Chilean SRI and the Spanish SRI lies in the Likert scale, which uses “totally disagree/totally agree” instead of “totally false/totally true”, in addition to including two local adaptations: an enlarged visual Likert scale for patients requiring assistance and read by the interviewer (approved in the semantic validation), and increased font size of the text (from size 10 to 14).The average time to complete the questionnaire was considered adequate for its clinical application, although it is greater than that needed in other face-to-face validations (36).The Chilean sample presented a lower percentage of self-administered tests relative to the total patients surveyed (46.8%; 116 of 248) compared to the Spanish sample (54%; 62 of 115) and the German sample (97%; 219 of 226), that is, the Chilean patients required more assistance from the interviewer.

The Chilean version of SRI showed good psychometric properties through good levels of internal consistency in its seven dimensions and very good temporal stability in the retest (Table 4).

The construct validity assessed by EFA presented very different results from the German SRI. In the latter, 59.8% of the total variance was explained by a single factor, thus analysis was not continued, and orthogonal rotation was not performed, ultimately selecting a single-factor model with a single summary scale and seven theoretical dimensions developed by the expert panel. The Spanish study, like the Chilean study, did not find in its sample a single-factor model, but rather extracted factors through orthogonal rotations that explained the model [14].

The Chilean study group rejected modifying the final structure of the questionnaire; that is, the seven original theoretical scales were not changed to eight according to the equimax rotation to avoid decreasing the internal consistency (alpha).

Analyzing the hypotheses, one of the most relevant findings in the Chilean sample is that patients with COPD did not present the worst scores on the Respiratory Complaints or Anxiety dimensions (as postulated by the original SRI) but rather the patients with TB sequelae (Table 5). This is explained by the level of severity of these patients, who in prior survival analyses had the worst survival since admission to the LTH-NIV [34].

Another finding was that the COPD group did not show a significant difference in Anxiety versus the OHS group (*p* = 0.53), differing from the German or Spanish samples. This difference may lie in underlying psychopathological disorders (depression and anxiety) associated with extreme obesity rather than having a relationship with deterioration in lung capacity.

Additionally, there was no significant difference in Physical Functioning between patients with neuromuscular and non-neuromuscular disease. This is likely related to the pulmonary and functional capacity characteristics of the Chilean patients, who showed greater deterioration than European groups given the delayed access to ventilatory treatment, which affected the Physical Functioning scores, which in turn were similar between patients with neuromuscular and non-neuromuscular diseases.

Last, in the total sample, there was no significant correlation between hours/day of ventilation and the total SRI score (summary scale). Differences were only found when breaking down the HRQL by dimension, and only Physical Functioning had a significant and inverse correlation and was affected by use > 9 h/day, with no significant correlation observed in the other six dimensions. This result suggests that patients in the Chilean sample are very similarly affected in the Psychosocial (AX, SF, SR and WB) and Symptoms (RC, AS) dimensions with respect to the number of hours connected to the ventilator.

## Conclusion

Evaluating HRQL is essential to monitor public and private health programs that provide LTH-NIV.

The Chilean version of the Severe Respiratory Failure Insufficiency questionnaire has good psychometric properties. It is a viable, valid, and reliable instrument to be applied in adults (> 20 years old) with CRF of various causes and was found to be sensitive to assess the underlying characteristics of the local population.

The present study conforms to international validation standards and, similar to the original instrument, is not designed to measure HRQL in subjects under invasive mechanical ventilation (tracheostomized).

Two local adaptations were proposed: an enlarged visual Likert scale for patients requiring assistance and read by the interviewer (approved in the semantic validation), and increased font size of the text (from size 10 to 14).

The research group hopes to contribute to the study and management of patients with CRF in LTH-NIV programs and collaborate in questionnaire validation in other Latin American countries.

## Supplementary Information

**Additional file 1**. Home mechanical ventilation technical standard -versions-2008-2012-and-2013 AND INFORMED CONSENT CHILE.

**Additional file 2**. Semantic validation of the SRI quality of life questionnaire 2021 Article Protocol SRI 1.0.

**Additional file 3**. Semántica Validación en Español del Cuestionario de Insuficiencia Respiratoria Severa en CHILE. Artículo Protocolo IRS 1.0.

## Data Availability

The datasets generated and /or analysed during the current study are not publicly available due to the ethical standards established by the law of duties and rights of patients Nº 20584 promulgated in 2012 by the Chilean State but are available from the corresponding author on reasonable request.

## Abbreviations

BMI: Body mass index
COPD: Chronic obstructive pulmonary disease
CRF: Chronic respiratory failure
EPAP: Expiratory positive airway pressure
FVC: Forced vital capacity
FEV_1_: Forced expiratory volume in 1s
HMV: Home mechanical ventilation
IPAP: Inspiratory positive airway pressure
IQR: Interquartile range
KYPH: Kyphoscoliosis
LTH-NIV: Long-term home non-invasive ventilation
MINREL: Ministry of Foreign Affairs of Chile
NMD: Neuromuscular disease
OHS: Obesity hypoventilation syndrome
PaCO2: Carbon dioxide arterial pressure
Non-CF BC: Non-cystic fibrosis bronchiectasis
TB: Pulmonary tuberculosis sequelae
SRI: Severe respiratory insufficiency questionnaire

## References

[1] Encuesta Nacional De Salud Del Año 2003. epi.minsal.cl/wp-content/uploads/2016/03/resumen-ejecutivo-vent.pdf

[2] MINSAL, Chile, Nacional ENS. Encuesta nacional de salud. Pontificia Universidad Católica de Chile, Universidad Alberto Hurtado, Chile 2009–2010. (Citado en agosto de 2017) disponible en: minsal.cl/ portal/url/item/bcb03d7bc28b64dfe040010165012d23. pdf

[3] Encuesta Nacional De Salud Del Año 2016–2017. https://www.minsal.cl/wp-content/uploads/2017/11/ENS-2016-17_PRIMEROS-RESULTADOS.pdf

[4] Menezes Am, Pérez-padilla R, Jardim Jr, Muiño A, López Mv, Valdivia G, Montes De Oca M, Talamo C, Hallal Pc, Victora Cg; Platino Team. Chronic Obstructive Pulmonary Disease In Five Latin American Cities (The Platino Study): A Prevalence Study. Lancet 2005; 366:1875–1881. 10.1016/S0140-6736(05)67632-5 16310554

[5] Carrillo A, Vargas R, Cisternas V, Olivares-tirado P (2017). Prevalencia de riesgo de apnea obstructiva del sueño en población adulta Chilena. Rev Chil Enferm Respir.

[6] Bergner M, Bobbitt RA, Carter WB, Gilson BS (1981). The sickness impact profile: development and final revision of a health status measure. Med Care.

[7] McHorney CA, Ware JE, Lu JF, Sherbourne CD (1994). The MOS 36-item short-form health survey (SF-36): III. tests of data quality, scaling assumptions, and reliability across diverse patient groups. Med Care.

[8] Jones PW (1991). Quality of life measurement for patients with diseases of the airways. Thorax.

[9] Guyatt G, Berman L, Townsend M, Pugsley S, Chambers L (1987). A measure of quality of life for clinical trials in chronic lung disease. Thorax.

[10] Garrod R, Bestall JC, Paul EA, Wedzicha JA, Jones PW (2000). Development and validation of a standardized measure of activity of daily living in patients with severe COPD: the London chest activity of daily living scale (LCADL). Respir Med.

[11] Sociedad Española de Neumología y Cirugía Torácica. Manual Separ De Procedimientos N°12: Herramientas para la Medida de la Calidad de Vida Relacionada con la Salud. 2007.

[12] Windisch W, Freidel K, Schucher B, Baumann H, Weibel M, Matthys H (2003). The severe respiratory insufficiency (SRI) Questionnaire: a specific measure of health-related quality of life in patients receiving home mechanical ventilation. J Clin Epidemiol.

[13] SRI Project; Deutsche Gesellschaft für Pneumologie und Beatmungsmedizin e.V. https://www.atemwegsliga.de/en-sri.html

[14] López-Campos J, Failde I, León Jiménez A, Masa Jiménez F, Barrot Cortés E, Benítez Moya JM (2006). Calidad de vida relacionada con la salud de pacientes en programa de ventilación mecánica domiciliaria La versión Española del cuestionario SRI. Arch Bronconeumol..

[15] Andrade M, Antolini M, Canales K, Fuentes M, Mazzei M, Maquilón C (2018). Caracterización socio-demográfica y clínica de pacientes adultos en ventilación mecánica no invasiva domiciliaria. Ministerio de Salud. Chile. Rev chil Enferm respir..

[16] Muñiz J, Elosua P, Hambleton RK (2013). Directrices para la traducción y adaptación de los tests: Segunda edición. Psicothema.

[17] Ramada-Rodilla JM, Serra-Pujadas C, Delclós-Clanchet GL (2013). Adaptación cultural y validación de cuestionarios de salud: revisión y recomendaciones metodológicas. Salud Pública Mex.

[18] Organization WHO; WHO translation & adaptation guidelines [Internet]. Available from: http://www.who.int/substance_abuse/research_tools/translation/en/#

[19] Wild D, Grove A, Martin M, Eremenco S, McElroy S, Verjee-Lorenz A, Erikson P (2005). Principles of good practice for the translation and cultural adaptation process for patient-reported outcomes (PRO) measures: report of the ISPOR task force for translation and cultural adaptation. Value Health.

[20] Sánchez R, Echeverry J (2004). Validación de escalas de medición en salud. Rev Salud Pública.

[21] Tornimbeni, S., Pérez, E., Olaz F. Introducción a la Psicometría: Confiabilidad. In: 1a Edición Buenos Aires; 2008. p. 71–96.

[22] Tornimbeni, S., Pérez, E., Olaz F. Introducción a la Psicometría: Validez. In: 1a Edición. Buenos Aires; 2008. p. 101–32.

[23] Morales-Vallejo P. El Análisis Factorial en la construcción e interpretación de tests, escalas y cuestionarios. Univ Pontif Comillas. 2011;45.

[24] Martínez CM, Alonso M, Sepúlveda R (2012). Metodología de Investigación: Introducción al Análisis factorial exploratorio. Rev Colomb Psiquiatría.

[25] Zamora Muñoz S, Monroy Cazorla L, Chavez Alvarez C. Análisis factorial: una técnica para evaluar la dimensionalidad de las pruebas. Cuaderno técnico 6. México, D.F; 2010. Report No.6

[26] Alvarado R, Pérez-franco J, Marchetti N, Aranda W (2012). Validación de un cuestionario para evaluar riesgos psicosociales en el ambiente laboral en Chile - ISTAS 21. Rev Med Chile.

[27] Alvarado R, Jadresic E, Guajardo V (2015). First validation of a Spanish-translated version of the Edinburgh postnatal depression scale (EPDS) for use in pregnant women. A Chilean study. Arch Women’s Mental Health.

[28] Lera L, Fuentes-García A, Sánchez H, Albala C (2013). Validity and reliability of the SF-36 in Chilean older adults: the ALEXANDROS study. Eur J Ageing.

[29] Olivares - Tirado, P. Estado De Salud De Beneficiarios Del Sistema De Salud De Chile: 2004 -2005, Departamento de Estudios y Desarrollo. disponible en sitio web: https://www.supersalud.gob.cl/documentacion/666/articles-1062_recurso_1.pdf

[30] Markussen H, Lehmann S, Nilsen RM, Natvig GK (2015). The Norwegian version of the severe respiratory insufficiency questionnaire. Int J Nurs Pract.

[31] Ghosh D, Rzehak P, Elliott MW, Windisch W (2012). Validation of the English severe respiratory insufficiency questionnaire. Eur Respir J.

[32] Ribeiro C, Ferreira D, Conde S, Oliveira P, Windisch W. Validation of the Portuguese Severe Respiratory Insufficiency Questionnaire for home mechanically ventilated patients. Rev Port Pneumol. 2017;23(3):139–145. 10.1016/j.rppnen.2017.01.001 28238622

[33] López-Campos JL (2008). Transculturally adapted Spanish SRI questionnaire for home mechanically ventilated patients was viable, valid, and reliable. J Clin Epidemiol.

[34] Maquilón C, Antolini M, Valdés N (2021). Results of the home mechanical ventilation national program among adults in Chile between 2008 and 2017. BMC Pulm Med.

