# Supplementary material for: Validation of the severe respiratory insufficiency questionnaire for Chile
Source: BMC Pulm Med. 2022 Jul 19;22:277. doi: 10.1186/s12890-022-02050-7 (PMC9295393; doi:10.1186/s12890-022-02050-7)
Supplement: Supplementary file 2 — Additional file 2. Semantic validation of the SRI quality of life questionnaire 2021 Article Protocol SRI 1.0. [file 12890_2022_2050_MOESM2_ESM.doc]

**Semantic Validation of the Severe Respiratory Insufficiency (SRI) quality of life questionnaire for Chilean adults on home non-invasive mechanical ventilation**

**Main author**

- Marianela Andrade Andrade, Kinesiologist, MSc. in Public Health, Kinesiologist Coordinator, Adult Home Ventilation Assistance Program, AVNIA MINSAL. marianelaandradea@gmail.com; cell: +56999111777

**Co-authors**

- Monica Antolini, Bronchopulmonary Physician, National Coordinator of the AVNIA Program
- Krishnna Canales Hernández, National Coordinator of AVNIA Program Nurses
- Cesar Maquilón Ortiz, Head of the Respiratory Diseases Department, Dávila Clinic
- Mauricio Fuentes A., Statistician, School of Public Health, University of Chile
- Marinella Mazzei P., Sociologist, School of Public Health, University of Chile.

**Corresponding author:**

- Cesar Maquilon O., MD; Pneumologist, Head of the Department of Respiratory Clinica Davila
- Mailing address: Recoleta 464, Edificio H, 6th floor, Clínica Dávila. Commune of Recoleta, Santiago, Chile. Postal mail 8431657.
- E-mail: [cmaquilon@davila.cl](mailto:cmaquilon@davila.cl).
- E-mail: [cmaquilon87@hotmail.com](mailto:cmaquilon87@hotmail.com)
- Cell phone: +56992268873

**Introduction:** Chronic global respiratory failure (CRF) is a complex and heterogeneous condition present in both pulmonary and extrapulmonary diseases. Individuals who suffer from it have a high rate of morbidity and mortality in conjunction with physical disabilities and associated psychosocial dysfunction, all of which cause a deterioration in their health-related quality of life (HRQL) and an increase in healthcare costs. Home non-invasive mechanical ventilation (LTH-NIV) is a cost-effective treatment, and it is important to evaluate the HRQoL of its users. The German Schwerer Respiratorischer Insuffizienz (SRI) questionnaire has been shown in international studies to be a specific instrument suitable for evaluating HRQoL in adults with CGRF. **Objective:** To validate and cross*-*culturally adapt the SRI instrument for Chilean adults with CRF who users of LTH-NIV are. **Materials and Methods:** This was a quantitative, prospective study. German to Spanish translation and back-translation and semantic validation were conducted. Work teams consisting of four professional translators, a committee of experts in the field and an editor in charge, plus the advice of the original author, were formed. In-person meetings and communication via email were held in stages to consolidate versions of the Chilean SRI. A pilot group of 15 stable chronic patients on LTH-NIV >3 months was formed, and their suggestions and difficulties were recorded. The history of semantic and syntax adjustments was tabulated in a matrix. **Results:** A 49-item Chilean version of the SRI questionnaire was obtained (with no item eliminations). The translation presented a low task level of difficulty and a high degree of naturalness, with a mean application time of 14.5 ± 6 min. The items were found to be "fully equivalent" (category A), with a content validity index = 1. **Conclusions:** The Chilean version of the SRI instrument is semantically equivalent to the original German version. It received the approval of clinical experts. It is adaptable to the target population while considering reading and writing limitations. The protocol followed in the present study conforms to international validation standards.

**Keywords:** quality of life, home mechanical ventilation, psychometry, prolonged ventilation

**Introduction**

Health-related quality of life (HRQL) has acquired greater relevance for the evaluation of health interventions since it incorporates the patient's participation into traditional biomedical measurements. There are several definitions and approaches to the concept of HRQL, and there is no consensus, but in general, they focus on "the impact that a disease and its consequent treatment have on the patient's perception of their well-being" (Patrick and Erickson 1993). Well-being corresponds to the person's subjective perception of various aspects, such as physical, psychological, and social function. Due to this subjective nature, HRQL has limitations, and controversy has been generated in this regard, which will not be addressed in this article.

In the field of respiratory medicine, the questionnaires that evaluate HRQL are focused more frequently on specific studies on smoking, chronic obstructive pulmonary disease (COPD) and bronchial asthma.

The development of instruments that evaluate HRQL is carried out mostly in English, and generally, the first translations into Spanish are carried out in Spain. The Spanish versions are typically used in Latin America without performing semantic adaptation and local validation, as recommended, thus generating bias in the results.

Chronic global respiratory failure (CRF) is a complex condition secondary to a wide range of diseases of pulmonary origin (e.g., COPD, tuberculosis, bronchiectasis) and extrapulmonary origin, such as some rib cage alterations (kyphoscoliosis, hypoventilation, obesity) and acute and slowly progressive neuromuscular diseases plus a miscellany of other conditions. Due to their functional deterioration, this group of subjects has a higher rate of morbidity and mortality and may need advanced healthcare resources in the long term, including support with home non-invasive ventilation. In the subgroup of subjects who suffer from CRF and require LTH-NIV, the evaluation of HRQL is particularly relevant. Since it is not possible to fully recover from the disease, interventions are focused on reducing symptoms and avoiding complications. For this reason, it is necessary to apply a specific instrument for this condition, since generic instruments such as the SF-36 questionnaire do not include aspects that affect daily life, being less sensitive, while specific instruments that focus on only a single respiratory disease [e.g., Saint George's Respiratory Questionnaire (SGRQ) for COPD] are not comparable and exclude other diseases in the group with CRF.

In this sense, the instrument that meets the technical and psychometric requirements to be used in these patients is the Schwerer Respiratorischer Insuffizienz [Severe Respiratory Insufficiency (SRI)] questionnaire created in Freiburg, Germany in 2003, by the research team of Wolfram Windisch MD. It has been used internationally and was translated into Spanish in Spain in 2005 by José Luis López-Campos MD. This questionnaire followed a rigorous process of elaboration and included more than ten different diagnoses when formulating the questions. The SRI has been applied in Chile since 2008, but because of the lack of local validation, the Spanish version shows biased results when evaluating subjects in LTH-NIV.

The information provided by instruments that evaluate HRQL in patients with CRF allows better decision-making in terms of the development of healthcare goals, planning and policies, allowing to adequately evaluate LTH-NIV therapies in Chile.

**Overall Research Objective**

Perform the validation and transcultural adaptation of the Schwerer Respiratorischer Insuffizienz (SRI) instrument for the Chilean adult population suffering from chronic global respiratory failure who are users of home non-invasive mechanical ventilation.

**Specific Objectives**

1. Translate the SRI quality of life questionnaire into Spanish.
2. Carry out the semantic and cultural adaptation of the SRI quality of life questionnaire to the Chilean context.

**Materials and Methods**

**Characteristics of the original SRI instrument**

The SRI is a questionnaire self-administered by LTH-NIV users (non-tracheostomized) that consists of 49 items (statements), grouped into seven dimensions (scales) that the subject rates according to their perception on a Likert scale considering their state of health during the last week. The Likert scale is divided into five levels: "totally false; quite false; partly true/partly false; quite true and totally true [totally false; mostly false; partly true/partly false; mostly true and totally true]". The seven dimensions are respiratory complaints (RC); physical functioning (PF); attendant symptoms and sleep (AS); social relationships (SR); anxiety (AX); psychosocial well-being (WB) and social functioning (SF).

Once the instrument is filled out, of its 49 items, 35 are recoded by inverting their value to leave all the items in the same direction. After recoding these items, the score of each dimension is obtained according to a mathematical transformation that uses in the numerator the mean value of the scores obtained for the items of the dimension minus 1, divided by the maximum distance of the Likert scale, that is, divided by four. The dimensions are expressed in percentages (0 to 100%).

[Dimension score = (Mean value of items - 1)/4 * 100]

Each dimension score can be obtained if at least half of the items are answered. The total score of the SRI called the SRI Summary Scale is obtained from the mean value of all dimensions. If the value of a dimension is missing, it is not possible to calculate the total score. Once the result is obtained, high values indicate a good quality of life, and low values indicate a poor quality of life.

The German SRI in its preparation showed good strength in its psychometric properties, and the reliability estimated by internal consistency (Cronbach's alpha) was high, above 0.70 in all dimensions, and greater than 0.80 in four of them.

**Expert committee**

A committee of experts was formed with cardiorespiratory health professionals selected for their expertise and background (two were bilingual Spanish-German doctors). An editor in charge and fieldwork coordinator was also selected.

The translators were recommended by the German Embassy in Chile, and this was done to have four translators, two native Chileans and two native Germans, who worked in parallel and blinded, without communication with each other.

Translation Department of the Ministry of Foreign Affairs of Chile (MINREL, for its acronym in Spanish).

**Translation, cultural adaptation, content validity and semantic validation of the instrument**

The methodology called translation and backtranslation, cultural adaptation and validation was used, suggested by Latin American health measurement guidelines along with recommendations by the WHO (45) (46), seeking to obtain a Chilean version of the German SRI that has conceptual and cultural equivalence in all its structure, maintaining correspondence with the original instrument and achieving the expected understanding by an educated Chilean subject , with a level of knowledge equivalent to 12 years of age. Direct authorization was obtained from the original author, Wolfram Windisch MD.

The direct translation of the German SRI into Spanish was carried out by two native Chilean translators; one of these translations was carried out by the Translation Department of the Ministry of Foreign Affairs of Chile (MINREL, for its acronym in Spanish), thus having an official character. Both translations were agreed upon through meetings between the editor and expert committee, resulting in the version called SRI.cl 1.0. Subsequently, version SRI.cl 1.0 was back translated into German by the two native German translators. These back-translations were reviewed by the two bilingual doctors of the expert committee and by the consulting translator to consolidate the synthesized back-translated version, called SRI.cl 1.1.

Version 1.1 of the back-translation was sent to the original author (Windisch MD, Germany) to assess the existence of semantic differences with respect to the original SRI; if there were differences, the expert committee and consulting translator adjusted them to achieved "full equivalence". For this purpose, the items were classified into three groups: (A) fully equivalent, (B) similar, but not fully equivalent or with some questionable expression, and (C) questionable equivalence or non-equivalent. This categorization was the same used in the Spanish translation of the SRI by the research group of José López Campos MD (Spain).

**Pilot test for semantic validation**

The understanding of the Chilean SRI instrument version 1.1 was investigated, for which a convenience sample of 15 individuals with different educational levels was selected to provide corrections, comments and suggestions on any aspect that hindered their understanding of the test. The items that were difficult to understand by 15% of the respondents, i.e., more than two subjects, were reviewed to consider the suggested changes. The inclusion criteria for the pilot test were a) having CRF diagnosed by a bronchopulmonary physician and documented with laboratory tests); b) in treatment with LTH-NIV for more than 30 days prior to the test; (c) able to read and write; and d) being in a stable phase of the disease (e.g., not having an infection or respiratory exacerbation that required management in emergency services during the last 30 days).

The pilot test was executed by two professionals with experience in the application of the test, and the information obtained from the subjects was tabulated and consolidated by the editor in charge. Instructions were given to the subjects in a standardized manner, and the application time of the instrument was recorded to determine its viability. Last, the areas of the questionnaire that were difficult to understand were identified by the respondents through a structured interview.

A summary of the translation and back-translation process is shown in Figure 1.


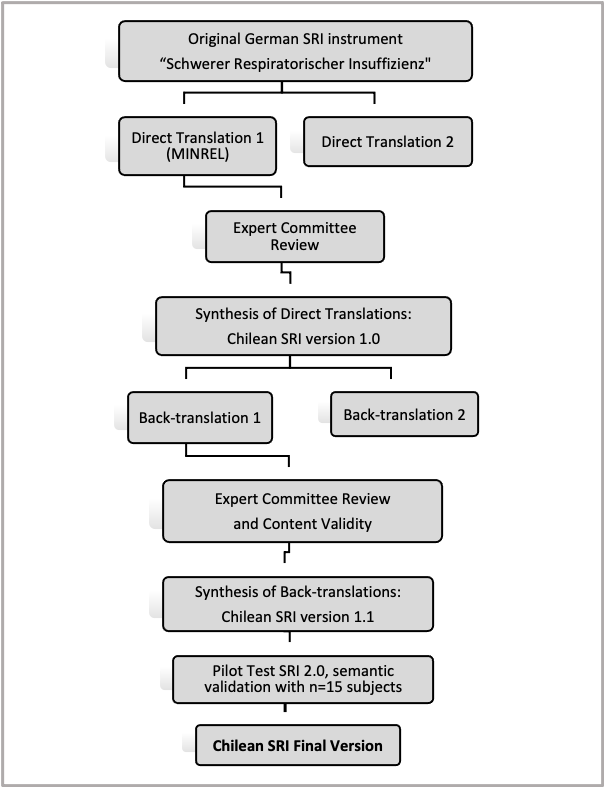


Figure 1. Diagram of the translation and back-translation process of the SRI into the Chilean version.

**Results**

**Translation and back-translation**

The two direct German-Spanish translations were equivalent in 44 of the 49 questionnaire items, and only 5 presented relevant differences (items 3, 15, 18, 25 and 47), which were adjusted. Of the 44 equivalent items, 8 items were identical in their translation, and the remaining 36 had minor differences in syntax (word arrangement) or synonyms that did not alter the content of the item; in these cases, the most relevant to the vocabulary of the instrument target was chosen. Version 1.0 of the Chilean SRI was rated by the translators with a difficulty level of 2 and a naturalness level of 9 (where 1 = minimum and 10 = maximum); that is, it was a translation with low difficulty and high naturalness.

Regarding the Likert scale, the two direct translations obtained were not equivalent and were not accepted by the expert committee (Table 1). The Likert scale of Translation 2 coincided to be the same type used by the Spanish SRI ("totalmente falso a totalmente verdadero [totally true or totally false]"), which, based on previous experience in Chilean subjects, has been difficult to understand, since stating true/false tends to be related to a dichotomous response. The expert committee established a new Likert scale whose options range from "totalmente en desacuerdo [strongly disagree]" to "totalmente de acuerdo [strongly agree]".

The title of the instrument "Schwerer Respiratorischer Insuffizienz (SRI)" was translated into "Severe Respiratory Failure Questionnaire (IRS)".

**Table**1. Selection process of the Likert Scale for the Chilean SRI.

| **Questionnaire** | **Likert scale response options** | | | | |
| --- | --- | --- | --- | --- | --- |
|  | Level -2 | Level -1 | Level 0 | Level +1 | Level +2 |
| **Original German SRI** | Trifft gar nicht zu | Trifft wenig zu | Trifft teils-teils zu | Trifft ziemlich zu | Trifft völlig zu |
| **SRI Direct translation 1** | It does not correspond at all | Not applicable | partly  Corresponds | Corresponds  pretty much | Absolutely corresponds |
| **SRI Direct translation 2** | Totally False | Quite False | Partly true/Partly false | Quite True | Totally true |
| **Expert Committee** | Strongly disagree | Disagree | Neither in agreement nor in disagreement | I agree | Totally agree |

In the versions back-translated into German, 15 identically translated items were obtained, and the remaining 34 presented minor differences in syntax, so the back-translations were expeditiously synthesized by the bilingual experts into one final version. The back translation was sent to the original author to assess the equivalence with the original SRI (Table 2), and according to this assessment, the following actions were taken: items that the author classified as type A remained unchanged; items classified as equivalence B and C were reviewed with the consulting translator and the bilingual doctors of the expert committee to adjust them or, on the contrary, to justify their maintenance to the original author given cultural equivalence.

Last, the corrected back-translation was again sent to the original author, who approved the 49 items, which were classified as category A. This allowed to subsequently analyse the cross-cultural adaptation of the items, information that was very useful for the final item consolidation step.

Among the psychosocial well-being (WB) domain items, there were cases that showed some difficulty in their cross-cultural adaptation. The adjustments made to the remaining items were minor and were typically related to the syntax of the statement or with semantic changes that did not alter their content.

**Table 2**. Summary of the equivalence rating of the items between the Chilean SRI and original German SRI given by the original author, Windisch MD.

| **Equivalence type items** | **Items** | **Number of Items** | **Determination of the committee of experts** |
| --- | --- | --- | --- |
| **Type A:** Fully equivalent | 1, 5, 6, 7, 9, 11, 17, 22, 24, 27, 29, 37, 38, 39, 41, 42, 43, 44, 49 | 19 | Did not require adjustments |
| **Type B:** Similar but not fully equivalent with some questionable expression | 2, 4, 8, 10, 12, 13, 14, 15, 16, 18, 19, 20, 21, 23, 25, 26, 28, 31, 32, 33, 34, 35, 40, 45, 46, 47, 48. | 27 | 14 of the 27 items were adjusted. Only Items 4, 25 and 28 required semantic adjustment |
| **Type C:** Questionable equivalence or non-equivalent | 3, 30, 36. | 3 | All 3 items were adjusted |

**Source:** self-made

Subsequently, the expert committee reviewed the worksheet with the history of the SRI versions obtained after the translation, back translation and assessment by the original author and evaluated the semantic and cultural differences and equivalences of the items. The result of the review was that 37 (75.5%) of the 49 items in this final step did not require changes nor were commented on, and only 12 items (24.5%) were adjusted, mainly based on background information compiled after the equivalence rating by the original author.

**Content validity**

The results of the evaluations by the experts were favourable; they considered the SRI an instrument that is methodologically well developed and that includes the domains required to evaluate subjects with CGRF. In addition, the four judges showed a high degree of agreement that the instrument items are pertinent, relevant and clear, giving an overall mean rating of 4.0 points (high level of compliance). The content validity index (CVI) of the instrument was = 1, so it had maximum agreement. Due to these results, the dimensions were maintained, and it was not proposed in this step to include or exclude items.

**Semantic validation and pilot test**

The pilot questionnaire of the Chilean SRI 2.0 was applied to a sample of 15 users of LTH-NIV belonging to the AVNIA MINSAL Program, residing in nine communes of the metropolitan region of Santiago, who were interviewed in their home by a nurse and a kinesiologist who had specific preparation for the execution of the test.

The sample consisted of 8 men and 7 women, whose mean age was 56.3 ± 20.5 years of different educational levels (Table 3), with an average permanence in the HNIMV programme of 3.2 ± 2 years. Of this sample, 9 subjects (60%) were oxygen dependent. The diagnoses causing respiratory failure were distributed as follows: COPD, n = 5 (33.3%); non-cystic fibrosis bronchiectasis, n = 1 (6.7%); neuromuscular disease n = 3 (20%); severe kyphoscoliosis, n = 3 (20%); and obesity hypoventilation syndrome, n = 3 (20%).

**Table 3**. Sociodemographic characteristics of the sample selected for the semantic validation pilot test of the Chilean SRI 2.0 (n = 15).

| Characteristics |  | n (15) | (%) |
| --- | --- | --- | --- |
| **Gender** | Male | 8 | 53.3 |
|  | Feminine | 7 | 46.7 |
| **Level of schooling** | Incomplete basic education | 3 | 20 |
|  | Complete basic education | 2 | 13.3 |
|  | Incomplete secondary education | 3 | 20 |
|  | Complete secondary education | 3 | 20 |
|  | Incomplete superior technique | 1 | 6.7 |
|  | Complete superior technique | 1 | 6.7 |
|  | Incomplete university | 1 | 6.7 |
|  | Complete university | 1 | 6.7 |
| **Marital status** | Single | 7 | 46.7 |
|  | Married | 4 | 26.7 |
|  | Divorced | 1 | 6.7 |
|  | Widower | 3 | 20 |
| **Employment situation** | Domestic activities | 2 | 13.3 |
|  | Student | 1 | 6.7 |
|  | Retire | 3 | 20 |
|  | Retired due to disability | 6 | 40 |
|  | Occasional or unstable work | 1 | 6.7 |
|  | Stable or employee work | 2 | 13.3 |

Thirteen participants self-administered the instrument and two requested it be read by the interviewer. They were given a sheet with the printed and enlarged Likert scale (Likert scale in laminated letter-size paper, Arial font size 24) to select their response. After answering the SRI 1.1, they were interviewed in a structured way to evaluate their understanding and record their comments about the vocabulary and wording used in all the components of the instrument: presentation, introduction to questions, Likert scale and items. The mean application time of the instrument was 14.5 ± 6 minutes. As a result, there were no comments on or changes suggested to the presentation text or introduction to items, and all participants reported good understanding. Regarding the Likert scale, two subjects reported difficulty in understanding the numerical value 0, "neither agree nor disagree", which required an explanation by the interviewer, which they understood; they were then asked if they preferred to change it and they answered "no". In regard to the 49 item statements, 44 elicited no comments and only 5 (10.2%) were changed, namely, items 9, 14, 17, 19 and 30. In item 9, "I fall asleep easily”, five subjects did not understand the word "conciliar", and after the interviewer explained the meaning, they preferred to change it to "Puedo fall asleep easily". For items 14, 17 and 19 that begin with the adverb “often have neck ache", "I often wake up at night" and "I often wake up at night", and "I get short of breath when I breathe"), seven respondents reported having a better understanding of "frequently [frequently]", which they considered simpler. The respondents were also asked their opinion regarding the use of an extended Likert scale for people with visual difficulties, which the 15 subjects approved and considered useful.

At the end of this process, the Chilean SRI version 2.0 was obtained with the 49 semantically validated items, which are presented in summary in Figure (the complete version of the questionnaire is shown in Figure 2).

**Recommendations for the application of the Chilean SRI based on local results**

For adult users of LTH-NIV in Chile, it is recommended, based on this protocol, to use the printed questionnaire with font size 14 (larger than the original, which is font size 11). In addition, it is recommended to attach an expanded visual Likert scale (Annex 1) to the questionnaire to be used by patients who have difficulty seeing or writing and need to indicate their preference manually.

It is recommended to evaluate HRQoL using the Chilean SRI questionnaire in all patients who use HNIMV, with a measurement taken before the intervention (baseline score without having started the use of the mechanical ventilator), and to subsequently apply it according to the doctor's plan.

**Discussion**

The objective of the present study was to obtain a specific quality of life questionnaire for the Chilean population of adults suffering from chronic global respiratory failure dependent on home non-invasive mechanical ventilation through the translation and validation of the German Severe Respiratory Insufficiency (SRI) questionnaire. A rigorous protocol was executed, performing all the steps according to the recommendations of the literature and selecting a study design with the same bases as the original instrument.

The strengths of this study include the authorization and assessment by the original author of the instrument, the participation of professional translators, even obtaining an official translation into Spanish (MINREL Chile), in addition to the establishment of a strong expert committee with accessible communication. Another strength was that the pilot test was carried out in person in the subjects' homes by respiratory health professionals.

The Chilean questionnaire presented a low level of difficulty in translation and a high degree of naturalness in its items, with an adequate mean application time for application at home or in a health care centre. The main difference of the Chilean version from the Spanish version is the selected Likert scale, since the Chilean version used the degree of agreement with the statement, "strongly disagree/strongly agree" versus "totally false/totally true" of the Spanish SRI. In addition, an expanded Likert scale was included as a support accessory for subjects who required assistance and reading by the interviewer. Finally, as a last local adaptation, it was determined to increase the font size of the questionnaire to size 14, which aims to accommodate the text reading limitation presented, considering that the target population is mainly older adults.

The sample highlights the social vulnerability of the group of respondents of the pilot test, expressed in their low education and work disability and unemployment status, parameters that are poorer compared to the group investigated in Spain.

It is important to note that the Chilean SRI questionnaire is the first validation carried out in Latin America and is expected to be useful for health teams in the region that promote home ventilation programmes since, as described, it is a cost-effective technology to manage patients with CGRF.

This article lays the foundation for conducting the psychometric evaluation of the Chilean SRI

1. I have a hard time climbing stairs.
2. I lack the air when eating.
3. I can go out at sunset.
4. I often feel down.
5. I also lack the air without physical exertion.
6. I often have a headache.
7. I have many friends and acquaintances.
8. I worry that my illness may get worse.
9. I can fall asleep easily.
10. I relate well with other people.
11. Sometimes I get dizzy.
12. At night I wake up with shortness of breath.
13. I'm afraid that at night I'm short of breath.
14. I often have pain in the back of my neck.
15. Illness forces me to stay at home.
16. I have a hard time doing things in the house.
17. I often wake up at night.
18. I can sleep around all night.
19. I often lack the air to breathe.
20. I look to the future with optimism.
21. I feel alone/alone.
22. I lack the air when I speak.
23. Visits exhaust me.
24. I cough a lot.
25. I often have my airways with phlegm.
26. I avoid situations where I can be ashamed of my breathing problems.
27. I feel good among my friends/acquaintances.
28. I am afraid of a chock attack.
29. I lack the air when making physical efforts.
30. The limitations of my illness bother me quite a bit.
31. My marriage/relationship has been affected by my illness.
32. I can go out and shop.
33. I can dedicate myself to the hobbies I like.
34. I get angry often.
35. Due to my illness, I have less contact with myfriends/acquaintances.
36. I'm enjoying my life.
37. I can participate in social activities.
38. I am often sad.
39. My breathing problems bother me in public.
40. I often get nervous.
41. I can dress alone.
42. I feel tired during the day.
43. I feel isolated.
44. I deal well with my illness.
45. My breathing problems hinder my daily activities.
46. My illness affects my family life.
47. I have lost contact with other people due to my breathing problems.
48. My possibilities for recreation are limited.
49. Overall, I'm happy with my life.

**Figure 2**. Final items of the Chilean SRI version 2.0 resulting from the semantic validation pilot test.

**Annex 1**. Expanded Likert Scale (support material for answering the Chilean SRI questionnaire)

| **Strongly disagree**  **-2** | **Disagree**  **-1** | | **Neither agree nor disagree**  **0** | | **Agree**  **+1** | | | **Strongly agree**  **+2** |
| --- | --- | --- | --- | --- | --- | --- | --- | --- |
| **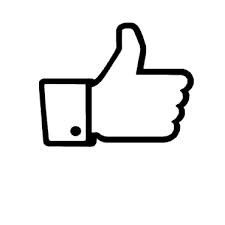** | |  | |  | |  | **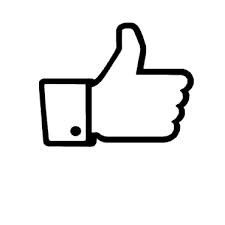** | |
