# Supplementary material for: Validation of the severe respiratory insufficiency questionnaire for Chile
Source: BMC Pulm Med. 2022 Jul 19;22:277. doi: 10.1186/s12890-022-02050-7 (PMC9295393; doi:10.1186/s12890-022-02050-7)
Supplement: Supplementary file 3 — Additional file 3. Semántica Validación en Español del Cuestionario de Insuficiencia Respiratoria Severa en CHILE. Artículo Protocolo IRS 1.0. [file 12890_2022_2050_MOESM3_ESM.docx]

**Protocolo de Validación del Cuestionario de Calidad de Vida “Severe Respiratory Insuficiency” (S.R.I.) para la población chilena de adultos en Ventilación Mecánica No Invasiva Domiciliaria.**

**Autor principal:**

- Marianela Andrade A, Kinesióloga, MsC Salud Publica, Coordinadora de Kinesiólogos Programa Asistencia Ventilatoria Domiciliaria Adultos, AVNIA MINSAL.

**Co Autores:**

- Mónica Antolini, Medico Broncopulmonar Coordinador Nacional Programa AVNIA
- Krishnna Canales Hernández, Coordinadora Nacional Enfermeros Programa AVNIA
- Cesar Maquilón Ortiz, jefe del Servicio de Enfermedades Respiratorias, Clínica Dávila
- Mauricio Fuentes A., Estadístico, Escuela de Salud Publica Universidad de Chile.

**Introducción:** La Falla respiratoria global crónica (FRGC), es una condición compleja y heterogénea presente tanto en patologías de origen pulmonar como extrapulmonar. Los sujetos que la padecen poseen una elevada tasa de morbimortalidad en conjunto con discapacidad física y disfunción psicosocial asociada, lo cual genera deterioro de su calidad de vida relacionada con la salud (CVRS) y costos sanitarios. La ventilación mecánica no invasiva domiciliaria (VMNID) es un tratamiento costo efectivo, siendo relevante evaluar la CVRS de sus usuarios. El cuestionario S.R.I. de origen alemán, ha demostrado en estudios internacionales ser un instrumento especifico adecuado para evaluar CVRS en adultos con FRGC. **Objetivo:** Realizar la validación y adaptación transcultural del instrumento “*Schwerer Respiratorischer Insuffizienz*, *(S.R.I.) “en* población chilena de adultos que padecen FRGC usuarios de VMNID.

**Material y método:** Estudio cuantitativo, prospectivo. Metodología Traducción- Retro traducción alemán español y Validación semántica. Se conformaron equipos de trabajo (4 traductoras profesionales, Comité de expertos en el área y un editor a cargo) más la asesoría del autor original. Se celebraron reuniones presenciales y comunicación vía correo electrónico por etapas para consolidar versiones del IRS chileno. Se conformó grupo piloto de 15 pacientes crónicos estable con VMNID >3 meses, se registran sus sugerencias y dificultades. Se tabulo en matriz el historial de ajustes semánticos y sintaxis. **Resultados:** Se logra cuestionario SRI Chileno, 49 ítems (sin eliminación), en la traducción presentó un bajo nivel de dificultad y un alto grado de naturalidad en sus ítems, con un tiempo medio de ejecución14,5 ± 6 min. Los ítems logran ser “totalmente equivalentes” (cat. A) Índice de Validez de contenido=1. **Conclusiones:** El instrumento SRI en su versión chilena es equivalente semánticamente con la versión original alemán. Cuenta con aprobación de expertos clínicos. Logra adaptarse a la población objetivo considerando sus limitaciones en lectoescritura. El protocolo seguido en la presente investigación se ajusta a los estándares internacionales de validación.

Palabras clave: calidad de vida, ventilación mecánica domicilio, psicometría, ventilación prolongada.

Email autor: marianelaandradea@gmail.com cel. +56999111777

**Introducción:**

La Calidad de vida relacionada a la salud (CVRS) ha adquirido mayor relevancia para la evaluación de intervenciones sanitarias ya que a las mediciones biomédicas tradicionales incorpora la participación del paciente. Para el concepto de CVRS existen varias definiciones y enfoques, no hay consenso, pero en general se centran en: “el impacto que una enfermedad y su consecuente tratamiento tienen sobre la percepción del paciente de su bienestar” (Patrick y Erickson 1993), este bienestar correspondería a la percepción subjetiva de la persona en diversos aspectos, tales como el físico, psicológico y la función social. Debido a este carácter subjetivo, la CVRS tiene limitaciones y se ha generado controversia al respecto, lo cual no será abordado en este artículo.

En el ámbito de la medicina respiratoria, los cuestionarios que evalúan CVRS se enfocan con mayor frecuencia en estudios específicos de tabaquismo, enfermedad pulmonar obstructiva crónica (EPOC) y asma bronquial.

La elaboración de instrumentos que evalúan la CVRS se realiza mayormente en idioma inglés y generalmente las primeras traducciones al castellano se realizan en España. En Latinoamérica habitualmente se utilizan las versiones españolas no realizándose la adaptación semántica y validación local, como es recomendado, generándose así sesgo en los resultados.

La falla respiratoria global crónica (FRGC) es una condición compleja, secundaria a una amplia gama de patologías no tan solo de origen pulmonar (ej. EPOC, tuberculosis, bronquiectasias), sino también extrapulmonar como algunas alteraciones de la caja torácica (Cifoescoliosis, hipoventilación obesidad) y enfermedades neuromusculares agudas y lentamente progresivas más una miscelánea de otras enfermedades. Debido a su deterioro funcional, este grupo de sujetos posee una mayor tasa de morbimortalidad y pueden necesitar recursos sanitarios avanzados a largo plazo, dentro de ellos el soporte con ventilación mecánica no invasiva domiciliaria (VMNID). En el subgrupo de sujetos que padecen FRGC y requieren VMNID evaluar la CVRS es particularmente relevante, ya que al no ser posible la recuperación total de su enfermedad, las intervenciones se concentran en disminuir los síntomas y evitar las complicaciones. Por esa razón es necesario aplicar un instrumento específico para dicha condición, ya que los instrumentos genéricos como por ejemplo el cuestionario SF-36 no incluyen aspectos que afectan su vida diaria, siendo menos sensibles y por el contrario, los instrumentos específicos que se enfocan sólo en una patología respiratoria (Ej.: Saint George´s SGRQ para EPOC) no son homologables y excluyen a otras enfermedades del grupo con FRGC.

En este sentido el instrumento que cumple los requisitos técnicos y psicométricos para ser utilizado en estos pacientes es el “Schwerer Respiratorischer Insuffizienz-SRI” **(**“Severe Respiratory Insufficiency- SRI Questionnaire” en ingles) (2) creado en Freiburg, Alemania el año 2003, por el equipo de investigación del Dr. Wolfram Windisch, ha sido usado internacionalmente y fue traducido al castellano en España el año 2005 (investigador José Luis López Campos) (3), este cuestionario siguió un riguroso proceso de elaboración e incluyó a más de diez diagnósticos diferentes al formular las preguntas. El IRS se aplica en Chile desde el 2008 pero en la versión española evaluando sujetos de los programas de VMNID, pero al no poseer la validación local posee sesgo al analizar sus resultados.

La información que entregan los instrumentos que evalúan la CVRS en enfermos con FRGC permite una mejor toma de decisiones en cuanto al desarrollo de objetivos, planificación y políticas sanitarias, permitiendo evaluar adecuadamente las terapias de VMNID en Chile.

**Objetivo General de Investigación**

Realizar la validación y adaptación transcultural del instrumento “*Schwerer Respiratorischer Insuffizienz*, *(S.R.I.)“* (26) para la población adulta chilena que padece de falla respiratoria global crónica usuaria de ventilación mecánica no invasiva domiciliaria.

**Objetivos Específicos**

1. Traducir al español el cuestionario de Calidad de Vida S.R.I.
2. Realizar la adaptación semántica y cultural del cuestionario de Calidad de Vida S.R.I. al contexto chileno.

**Método**

**Características del Instrumento SRI original**

Es un cuestionario auto aplicado, realizado a usuarios de VMNID (no traqueostomizados) que consta de 49 ítems (afirmaciones), agrupadas en siete dimensiones (escalas) que el sujeto califica según su percepción en la escala Likert considerando su estado de salud durante la última semana. La escala Likert esta graduada en cinco niveles: “totalmente falso; bastante falso; en parte verdadero/en parte falso; bastante verdadero y totalmente verdadero” (según traducción española). Las siete dimensiones son: Síntomas Respiratorios (SR); Función Física (FF); Síntomas Acompañantes y Sueño (SS); Relaciones Sociales (RS); Ansiedad (AX); Bienestar Psicosocial (BP) y Función Social (FS).

Una vez ejecutado el instrumento, de sus 49 ítems, 35 se recodifican invirtiendo su valor, para dejar todos los ítems en el mismo sentido. Tras recodificar estos ítems, la puntuación de cada dimensión se obtiene según una transformación matemática que utiliza en el numerador el valor promedio de los puntajes obtenidos para los ítems de la dimensión menos 1, dividido por el máximo recorrido de la escala Likert, es decir dividido por cuatro. Las dimensiones se expresan en porcentajes (0 a 100%)

[Puntaje de la Dimensión= (Valor medio de ítems -1) /4 * 100]

Cada puntaje de la dimensión se puede obtener si al menos se cuenta con la mitad de los ítems respondidos. El puntaje total del IRS llamado “IRS Escala Suma” se obtiene del valor promedio de todas las dimensiones. Si faltase el valor de una dimensión, no es posible calcular el puntaje total. Una vez obtenido el resultado, los valores altos indican una buena calidad de vida, y los bajos, una mala calidad de vida.

El SRI alemán en su elaboración demostró tener buena fortaleza en sus propiedades psicométricas, la confiabilidad estimada mediante consistencia interna (alfa de Cronbach) fue alta 0.70 en todas las dimensiones, logrando más que 0,80 en cuatro de ellas.

**Comité de expertos**

Se conformó un comité de expertos con profesionales de salud cardiorrespiratoria, seleccionados por su expertiz y trayectoria (dos de ellos médicos bilingües español-alemán). Se definió un editor a cargo y coordinador de trabajo de campo.

Las traductoras recomendadas por la Embajada de Alemania en Chile (31) y se cumplió así con el fin de tener cuatro traductoras dos nativas chilenas y dos nativas alemanas, quienes trabajaron en forma paralela y ciega sin comunicación entre sí.

Departamento de Traducciones del Ministerio de Relaciones Exteriores de Chile (MINREL).

**Proceso de traducción, adaptación cultural del instrumento, validez de contenido y validación semántica**.

Se utilizó la metodología denominada Traducción-Retro traducción(42)(43)(44), adaptación cultural y validación sugerida por guías hispanoamericanas de medición en salud (42)(43)(44) junto con recomendaciones de la O.M.S. (45) (46), buscando obtener una versión chilena del S.R.I. alemán que tuviera equivalencia conceptual y cultural en toda su estructura, manteniendo la correspondencia con el instrumento original y logrando la comprensión esperada para un sujeto chileno escolarizado, con conocimiento equivalente a doce años de edad (44). Se obtuvo autorización directa del autor original Dr. Wolfram Windisch.

La traducción directa del SRI alemán al castellano, fue realizada por dos traductoras nativas chilenas, una de estas traducciones se realizó por el Departamento de Traducciones MINREL, teniendo carácter de oficial. Ambas traducciones se consensuaron a través de reuniones entre editor y comité expertos, logrando la versión denominada “IRS.cl 1.0”. Posteriormente la versión 1.0 IRS.cl se tradujo inversamente llevándola de vuelta al alemán, labor realizada por las dos traductoras nativas alemanas, dichas retro traducciones logradas se revisaron con los dos médicos bilingües del comité de expertos y el traductor asesor, para lograr consolidar la versión de síntesis de retro traducción o versión “IRS.cl 1.1”.

La versión 1.1 de retro traducción obtenida fue enviada al autor original (Dr. Windisch, Alemania) para que calificara la existencia de diferencias semánticas respecto del SRI original y de existir el comité de expertos y la traductora asesora las ajustarían para llevarlas a “equivalencia total”. Para ello los ítems se calificaron en tres grupos: (A) Totalmente equivalentes (B) Similar, pero no totalmente equivalente o con alguna expresión dudosa, y (C) De equivalencia dudosa o no equivalente. Esta categorización fue la misma forma utilizada en el proceso de traducción al español del S.R.I. por el grupo de estudio del Dr. José López Campos (España).

**Prueba Piloto para Validación Semántica**

Se estudió la comprensión del instrumento IRS chileno versión 1.1, para ello se seleccionó una muestra por conveniencia de quince personas de diferente nivel educacional (45)(42) para que emitieran correcciones, comentarios y sugerencias de cualquier aspecto que dificultase su comprensión de la prueba. Los ítems que obtuvieran dificultad de comprensión en el 15% de los entrevistados, es decir en más de 2 sujetos, serian revisados para considerar las modificaciones sugeridas (44). Los criterios de inclusión para el Piloto fueron: a) Padecer FRGC diagnosticada por medico broncopulmonar y documentada con exámenes de laboratorio); b) Estar en tratamiento con VMNID mas de 30 días previos al test; c) Que puedan leer y escribir; d) Estar en fase estable de su enfermedad (ej.: no estar cursando infección ni exacerbación respiratoria que haya requerido manejo en servicios de urgencia durante los ultimo 30 días).

La prueba piloto se ejecutó por 2 profesionales con experiencia en la aplicación de la prueba, la información obtenida de los sujetos fue tabulada y consolidada por el editor del estudio. De forma estandarizada se entregaron las instrucciones a los sujetos, se tomó el tiempo de aplicación del instrumento para verificar la viabilidad y finalmente mediante una entrevista estructurada se le consulto cuales fueron las áreas del cuestionario que presentaron dificultad para su comprensión.

A modo de resumen el proceso de traducción retro traducción se expone en la figura 1.

Figura 1. Esquema del proceso de traducción retro traducción IRS versión chilena.

**Resultados:**

**Traducción y retro traducción**

Las dos traducciones directas alemán-castellano, fueron equivalentes en 44 de los 49 ítems del cuestionario, sólo 5 presentaron diferencias relevantes (ítems nº 3, 15, 18, 25 y 47) las que fueron ajustadas. De los 44 ítems equivalentes, 8 ítems resultaron idénticos en su traducción y los 36 restantes presentaron diferencias menores en la sintaxis (organización de palabras) o sinónimos que no alteraban el contenido del ítem, en estos casos se eligieron los más atingentes al vocabulario del destinatario del instrumento. La versión 1.0 del IRS chileno lograda fue calificada por las traductoras con nivel de dificultad 2 y nivel de naturalidad logrado 9 (donde 1= mínimo y 10= máximo), es decir, fue una traducción con baja dificultad y alta naturalidad.

En relación con la escala Likert, destacó que las dos traducciones directas logradas no fueron equivalentes y no fueron aceptadas por el comité de expertos (Tabla 1). La escala Likert de la Traducción 2 coincidió ser el mismo tipo que utiliza el IRS España (“totalmente falso a totalmente verdadero”) la cual, con la experiencia previa en sujetos chilenos, ha presentado dificultad en su comprensión, ya que al enunciar verdadero/falso tiende a ser relacionada con una respuesta dicotómica. El comité de expertos estableció una nueva escala Likert cuyas opciones van de “Totalmente en desacuerdo” a “totalmente de acuerdo”.

El título del instrumento “Schwerer Respiratorischer Insuffizienz (SRI)”, se tradujo a “Cuestionario Insuficiencia Respiratoria Severa (IRS)”, denominación que usaremos de esta sección en adelante.

Tabla 1. Proceso de selección de la Escala Likert para el IRS chileno.

| **Cuestionarios** | **Opciones de respuesta Escala Likert** | | | | |
| --- | --- | --- | --- | --- | --- |
|  | Nivel -2 | Nivel -1 | Nivel 0 | Nivel +1 | Nivel +2 |
| **S.R.I. Original Alemán** | Trifft gar nicht zu | Trifft wenig zu | Trifft teils-teils zu | Trifft ziemlich zu | Trifft völlig zu |
| **IRS Traducción directa 1** | No corresponde en absoluto | No corresponde | En parte  corresponde | Corresponde  bastante | Corresponde absolutamente |
| **IRS Traducción directa 2** | Totalmente Falso | Bastante Falso | En parte verdadero /  En parte falso | Bastante Verdadero | Totalmente verdadero |
| **Comités expertos** | Totalmente en desacuerdo | En desacuerdo | Ni en acuerdo ni en desacuerdo | De acuerdo | Totalmente de acuerdo |

En las versiones de retro traducción al alemán, se obtuvieron 15 ítems traducidos idénticos y los 34 restantes presentaron diferencias menores de sintaxis, por ello la versión final de “síntesis de retro traducción” fue unificada de forma expedita por los expertos bilingües. La retro traducción, fue enviada al autor original para calificar la equivalencia con el SRI original (Tabla 2) y según la calificación se realizaron las siguientes acciones en: los ítems que el autor calificó como tipo A se mantuvieron sin cambios y los calificados como equivalencia B y C se revisaron con el traductor asesor y los médicos bilingües del comité de expertos, para ajustarlos o por el contrario justificar su mantención ante el autor dada equivalencia cultural.

Finalmente, la retro traducción corregida fue nuevamente enviada al autor original, quien aprobó los 49 ítems, siendo calificados como categoría A, permitiendo posteriormente analizar los ítems de forma transcultural, información que fue muy útil para la fase de consolidación final de los ítems.

En el ámbito de los ítems de Bienestar Psicosocial (BP) se presentaron casos con cierta dificultad en su adaptación cultural. Respecto de los ajustes realizados a ítems restantes, estos fueron menores, teniendo más relación con la sintaxis de la afirmación o con cambios semánticos que no alteraban

su contenido.

Tabla 2. Resumen de la calificación de Equivalencia de ítems, entre IRS chileno y original alemán, realizada por autor original Dr. Winsdich.


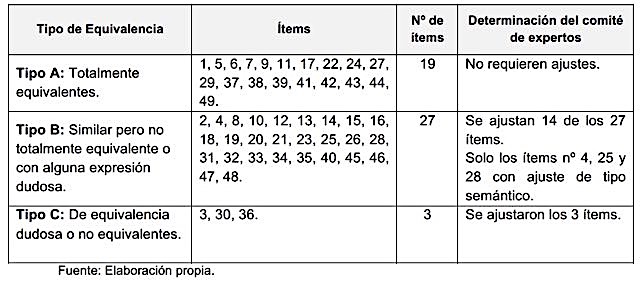


Posteriormente, el grupo de expertos revisó la planilla de trabajo con el historial de versiones IRS logradas en la traducción, retro traducción y la calificación del autor original, se evaluaron las diferencias y equivalencias semánticas y culturales de los ítems. El resultado de la revisión fue que 37 (75,5%) de los 49 ítems en esta fase final no requirieron modificaciones ni tuvieron observaciones, solo 12 ítems (24,5%) fueron ajustados, basados principalmente en antecedentes recopilados tras la calificación de equivalencia del autor.

**Validez de Contenido**

Los resultados de las evaluaciones de los expertos fueron favorables, consideraron al IRS un instrumento que metodológicamente está bien elaborado y que incluye los dominios requeridos a evaluar en sujetos con FRGC, además los cuatro jueces presentaron un alto grado de acuerdo respecto de que los ítems del instrumento son pertinentes, relevantes y claros, dando promedios generales de evaluación de 4.0 puntos (alto nivel de cumplimiento). El índice de validez de contenido (IVC) del instrumento fue =1, por lo que es acuerdo máximo, debido a estos resultados se mantuvieron las dimensiones y no se planteó en esta fase incluir o excluir ítems.

**Validación Semántica y Prueba Piloto**

Se aplicó el cuestionario piloto del IRS chileno 2.0 a una muestra de quince sujetos usuarios de VMNID pertenecientes al Programa AVNIA MINSAL residentes en nueve comunas de la región metropolitana de Santiago, los cuales fueron entrevistados en su hogar por una enfermera y una kinesióloga, quienes poseían preparación específica para la ejecución de la prueba.

La muestra fue conformada por 8 hombres y 7 mujeres, cuya edad media fue 56,3 ± 20,5 años, de diferente nivel educacional descrito en la Tabla 3, con una permanencia promedio en el programa de VMNID de 3,2 ±2 años. De esta muestra 9 sujetos (60%) eran oxigeno dependientes. Los diagnósticos causantes de la insuficiencia respiratoria se distribuyeron en EPOC n=5 (33,3%); Bronquiectasias No Fibrosis Quística n=1 (6,7%); Enfermedad Neuromuscular n=3 (20%), Cifoescoliosis severa n=3 (20%) y Síndrome Hipoventilación Obesidad n=3 (20%).

Tabla 3. Características sociodemográficas de la muestra seleccionada para Piloto de Validación Semántica del IRS 2.0 chileno (n=15)


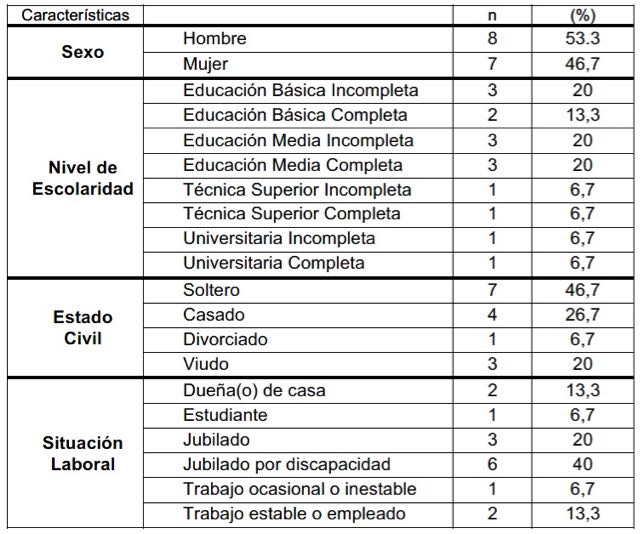


Trece participantes ejecutaron el instrumento de forma auto administrada y 2 solicitaron lectura por el entrevistador, a ellos se le entrego una cartilla con la escala Likert impresa y ampliada (escala Likert en hoja tamaño carta plastificada, fuente Arial tamaño 24) para que seleccionaran su respuesta. Luego de ejecutar el IRS 1.1. fueron entrevistados de forma estructurada para evaluar su comprensión y registrar los comentarios acerca del vocabulario y redacción utilizada en todos los componentes del instrumento: presentación, introducción a preguntas, escala Likert e ítems. El tiempo de administración del instrumento fue en promedio de 14,5 ± 6 minutos. Como resultado, la evaluación del texto de presentación e introducción a ítems no tuvieron observaciones ni modificaciones, todos los participantes refirieron buena comprensión. Respecto de la Escala Likert dos sujetos refirieron dificultad en comprender el valor numérico 0 de la escala “Ni en acuerdo ni en desacuerdo”, el cual requirió explicación por el entrevistador la cual entendieron, se les consultó si preferían cambiarla y respondieron que no. En relación con los 49 ítems de afirmaciones, 44 no tuvieron observaciones y sólo 5 (10,2%) fueron modificados, los que correspondieron al ítem nº 9, 14, 17, 19 y 30. En el ítem nº 9 “Puedo conciliar bien el sueño” cinco sujetos no comprendieron la palabra “conciliar”, y luego de que el entrevistador le explicara el significado prefirieron modificar por “puedo quedarme dormido fácilmente”. Para los ítems 14, 17 y 19 que inician con la locución adverbial “a menudo” (“A menudo me despierto por la noche” y “A menudo me falta el aire para respirar”) siete entrevistados refirieron entender mejor “con frecuencia”, considerado más simple. Se les consultó a los sujetos su parecer respecto del uso de una escala Likert ampliada para personas con dificultades visuales a lo cual los quince sujetos la aprobaron y consideraron útil.

Al termino de este proceso se obtuvo el IRS versión chilena 2.0 del instrumento con los 49 ítems validados semánticamente, los que se exponen en resumen en la Figura (la versión completa del cuestionario se adjunta en Figura 2.).

**Recomendaciones para la aplicación del IRS chileno en base a los resultados locales**.

Para los adultos usuarios de ventilación mecánica no invasiva domiciliaria (VMNID) en Chile, se recomienda en base al presente protocolo utilizar el cuestionario impreso con tamaño de fuente 14 (mayor al original que es en tamaño de fuente 11), además adjuntar a este cuestionario una escala visual Likert ampliada Anexo 1, para ser utilizada en caso de pacientes que tengan dificultad para ver, escribir y requieran señalar su preferencia de forma manual.

Se recomienda evaluar CVRS mediante el cuestionario IRS chileno a todo paciente que utilice VMNID, con una medición antes de la intervención (registro basal sin haber iniciado el uso del ventilador mecánico), y posteriormente aplicarlo según plan del médico o programa responsable.

**Discusión**

El objetivo del presente estudio fue obtener un cuestionario de calidad de vida específico para la población chilena de adultos que padecen falla respiratoria crónica global dependientes de ventilación mecánica no invasiva domiciliaria, mediante la validación del instrumento “*Severe Respiratory Inssuficiency Questionnaire (SRI)”* de origen alemán. Se ejecutó un protocolo riguroso, realizándose todos los pasos según las recomendaciones de la literatura y realizando un diseño de estudio con las mismas bases que el instrumento original.

Dentro de las fortalezas de esta investigación se encuentra la autorización y evaluación del autor original del instrumento, además de contar con la participación de traductoras profesionales, obteniendo incluso una traducción oficial al castellano (MINREL Chile), además se contó con un fuerte comité de expertos con comunicación accesible. Otra fortaleza fue, que la prueba piloto haya sido ejecutada de forma presencial en los domicilios, por profesionales de la salud respiratoria.

El cuestionario chileno logrado presentó en la traducción un bajo nivel de dificultad y un alto grado de naturalidad en sus ítems, con un tiempo medio de ejecución adecuado para ser aplicado en el domicilio o en un centro de salud. La principal diferenciación de la versión chilena respecto de la española fue la escala Likert seleccionada, ya que la versión chilena utilizó el grado de acuerdo con la afirmación, “totalmente en desacuerdo / totalmente de acuerdo” versus “totalmente falso/ totalmente verdadero” de IRS español. Además, se incluyó como accesorio de apoyo una escala Likert ampliada anexa para los sujetos que requieran asistencia y lectura por entrevistador. Finalmente, como última adaptación local, se determinó aumentar el tamaño de fuente del cuestionario entre (tamaño 14) lo cual pretende dar respuesta a la limitación que presentaron en la lectura del texto considerando que la población de destino es principalmente adulto mayor.
Destacan la muestra la vulnerabilidad social de un grupo de encuestados de la prueba piloto expresada en su baja escolaridad situación de incapacidad laboral y desempleo, parámetros más deteriorados respecto del grupo investigado en España.

Es importante señalar que el cuestionario IRS chileno es la primera validación realizada en Latinoamérica y se espera que sea útil para los equipos de salud de la región que impulsan programas de ventilación domiciliaria ya que como se ha descrito es una tecnología costo efectiva para manejar pacientes con FRGC.
El presente artículo sienta la base para desarrollar la evaluación psicométrica del IRS chileno.

1. Me cuesta subir escaleras.
2. Me falta el aire al comer.
3. Puedo salir al atardecer.
4. Con frecuencia me siento decaído.
5. También me falta el aire sin hacer esfuerzo físico.
6. Con frecuencia tengo dolor de cabeza.
7. Tengo muchos amigos y conocidos.
8. Me preocupa que mi enfermedad pueda empeorar.
9. Puedo quedarme dormido fácilmente.
10. Me relaciono bien con otras personas.
11. A veces me mareo.
12. Por las noches me despierto con falta de aire.
13. Tengo miedo de que por las noches me falte el aire.
14. Con frecuencia tengo dolor en la nuca.
15. La enfermedad me obliga a quedarme en la casa.
16. Me cuesta trabajo hacer las cosas de la casa.
17. Con frecuencia me despierto por la noche.
18. Puedo dormir de corrido toda la noche.
19. Con frecuencia me falta el aire para respirar.
20. Veo el futuro con optimismo.
21. Me siento solo / sola.
22. Me falta el aire al hablar.
23. Las visitas me agotan.
24. Toso mucho.
25. Con frecuencia tengo mis vías respiratorias con flema.
26. Evito situaciones en las que puedo pasar vergüenza por mis problemas para respirar.
27. Me siento bien entre mis amigos / conocidos.
28. Tengo miedo a sufrir un ataque de ahogo.
29. Me falta el aire al hacer esfuerzos físicos.
30. Las limitaciones de mi enfermedad me molestan bastante.
31. Mi matrimonio / relación de pareja se ha visto afectado/a por mi enfermedad.
32. Puedo salir a comprar.
33. Puedo dedicarme a los pasatiempos que me gustan.
34. Me enojo con frecuencia.
35. Debido a mi enfermedad tengo menos contacto con mis amigos / conocidos.
36. Estoy disfrutando mi vida.
37. Puedo participar en actividades sociales.
38. Con frecuencia estoy triste.
39. Mis problemas para respirar me molestan en público.
40. Con frecuencia me pongo nervioso/a.
41. Puedo vestirme solo/a.
42. Me siento cansado/a durante el día.
43. Me siento aislado/a.
44. Me las arreglo bien con mi enfermedad.
45. Mis problemas respiratorios dificultan mis actividades diarias.
46. Mi enfermedad afecta mi vida familiar.
47. He perdido el contacto con otras personas debido a mis problemas respiratorios.
48. Mis posibilidades de recreación están limitadas.
49. En general estoy contento con mi vida.

Figura 2. Ítems finales de IRS chileno versión 2.0 como resultado de Piloto de Validación Semántica.

Anexo 1. Escala Likert Ampliada (material de apoyo para responder a cuestionario IRS Chile)

| **Totalmente en desacuerdo**  **-2** | **En desacuerdo**  **-1** | | **Ni en acuerdo ni en desacuerdo**  **0** | | **De acuerdo**  **+1** | | | **Totalmente de acuerdo**  **+2** |
| --- | --- | --- | --- | --- | --- | --- | --- | --- |
| **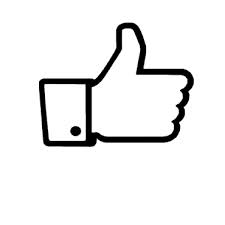** | |  | |  | |  | **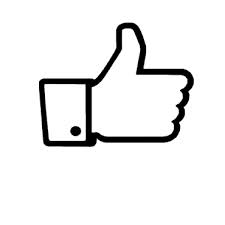** | |
